# Visceral Leishmaniasis in Ethiopia: An Evolving Disease

**DOI:** 10.1371/journal.pntd.0003131

**Published:** 2014-09-04

**Authors:** Samson Leta, Thi Ha Thanh Dao, Frehiwot Mesele, Gezahegn Alemayehu

**Affiliations:** 1 Adami Tullu Research Center, Ziway, Ethiopia; 2 National Institute of Veterinary Research, No 86, Hanoi, Vietnam; 3 Semera University, Semera, Ethiopia; New York University, United States of America

## Abstract

Visceral leishmaniasis (also known as kala-azar) is classified as one of the most neglected tropical diseases. It is becoming a growing health problem in Ethiopia, with endemic areas that are continually spreading. The annual burden of visceral leishmaniasis (VL) in Ethiopia is estimated to be between 4,500 and 5,000 cases, and the population at risk is more than 3.2 million. There has been a change in the epidemiology of VL in Ethiopia. Over the last decades, almost all cases and outbreaks of VL were reported from arid and semi-arid parts of the country; however, recent reports indicated the introduction of this disease into the highlands. Migration of labourers to and from endemic areas, climatic and environmental changes, and impaired immunity due to HIV/AIDS and malnutrition resulted in the change of VL epidemiology. HIV spurs the spread of VL by increasing the risk of progression from asymptomatic infection towards full VL. Conversely, VL accelerates the onset of AIDS. In Ethiopia, VL epidemiology remains complex because of the diversity of risk factors involved, and its control is becoming an increasing challenge. This paper reviews the changes in epidemiology of VL in Ethiopia and discusses some of the possible explanations for these changes. The prospects for novel approaches to VL control are discussed, as are the current and future challenges facing Ethiopia's public health development program.

## Introduction

Leishmaniases are a group of diseases caused by more than 20 species of the protozoan genus *Leishmania* that are transmitted between humans and other mammalian hosts by phlebotomine sandflies [1]. The disease endangers some 350 million people in 98 countries, most of them in the poorer regions of the globe [2], [3]. There are two major clinical forms of leishmaniasis, cutaneous leishmaniasis (CL) and visceral leishmaniasis (VL) [2]. VL is the most severe form of leishmaniasis, almost always fatal if untreated [4], . An estimated 200,000 to 400,000 new cases of VL occur worldwide each year, and from this, greater than 90% of VL human cases occur in six countries, namely Bangladesh, Brazil, Ethiopia, India, South Sudan, and Sudan [5]. Eastern Africa has the second highest number of VL cases, after the Indian Subcontinent. The disease is endemic in Eritrea, Ethiopia, Kenya, Somalia, Sudan, South Sudan, and Uganda [6], [7].

In Ethiopia, the first case of VL was documented in 1942 in the lower Omo plains, the southwestern part of the country [8]. The disease has spread to become endemic in many parts of the country. The disease is prevalent mostly in lowland, arid areas, and the parasite involved is mainly *Leishmania donovani*, with an estimated annual incidence of more than 4,000 cases [9]. Most important endemic foci include the Humera and Metema plains in the northwest [10], the Omo plains, the Aba Roba focus, and the Weyto River Valley in the southwest [11], [12].

In Ethiopia, VL mainly occurs in the arid and semi-arid areas; however, recent reports indicate spreading of the disease to areas where it was previously non-endemic [6], [13]–[16]. In 2003, an outbreak of VL occurred in highland areas of the Libo Kemkem district, in the Amhara regional state [16]. This is the only recorded VL outbreak from highland areas in Ethiopia; however, Ashford et al. [17] reported a few cases of VL in Belessa, a highland area in the Amhara regional state in the 1970s.

VL is becoming a growing public health threat; the spatial distribution and burden of VL is upsurging year after year [18]. VL–HIV co-infection is rising in Ethiopia, and it poses a new and difficult challenge to VL control effort. VL–HIV co-infection is characterized by a number of complexities, including challenging diagnosis, increased drug toxicity, and poor treatment response. Leishmaniasis in Ethiopia was formerly overseen by the Ministry of Health (MoH), but after the MoH underwent a large reorganisation in 2007, a national leishmaniasis task force was established with the aim of eliminating VL by 2015. Efforts made by the MoH so far to control VL are not withstanding its upsurge and, hence, VL is developing both on a spatial and temporal basis. Considering its recent upsurging, we thoroughly reviewed and analysed the previous works done, and we also propose a way forward to tackle this disease.

## Visceral Leishmaniasis in Ethiopia

### 1.1. Etiology

Leishmaniasis is caused by obligate intracellular protozoa of the genus Leishmania, belonging to the family *Trypanosomatidae* (order Kinetoplastida) [19], [20]. Human infection is caused by about 21 of 30 species that infect mammals. *Leishmania donovani complex* (*L. donovani* and *Leishmania infantum*) are the causative agents of VL in Ethiopia. *L. donovani* is regarded as the major cause of VL in Ethiopia, although *L. infantum* was identified as a main causative agent of the recent VL outbreak in Libo Kemkem, in the Amhara regional state [6], [16].

### 1.2. Hosts

Man and dogs are the most commonly affected hosts, and they can also be a potential reservoir [19], [20]. Dogs are the most important species among domesticated animals in the epidemiology of this disease, hence, dogs are reservoir hosts for *L. infantum*, which is one of the two most important organisms in human VL [19], [20]. According to Dereure et al. [21], domestic dogs might be the most important reservoir of *L. donovani* in eastern Africa. Some recent studies conducted by Bashaye et al. [22] in northern Ethiopia and Hassan et al. [23] in eastern Sudan also reported isolation of *L. donovani complex* from dogs. Moreover, Hassan et al. [23] reported highly significant attraction of different sandfly species to dog-baited traps. On the other hand, in East Africa, the transmission of *L. donovani* (the predominant causative agent of VL in the region) is thought to be anthroponotic, indicating that humans are a potential reservoir host for the disease [24]. Hence, the definitive reservoir of VL in East Africa in general and Ethiopia in particular remains to be determined.

A previous study conducted to determine the natural blood meal source of the sandfly in northern Ethiopia revealed a remarkably high bovine blood feed. The ELISA-based blood meal analysis of 273 fresh fed *Phlebotomus orientalis* females collected from Metema showed 92% of their blood meal source to be from bovine origin [25]. However, epidemiological significance of bovine in the transmission of leishmaniasis has not yet been investigated.

### 1.3. Life cycle and transmission

**Figure 1:**
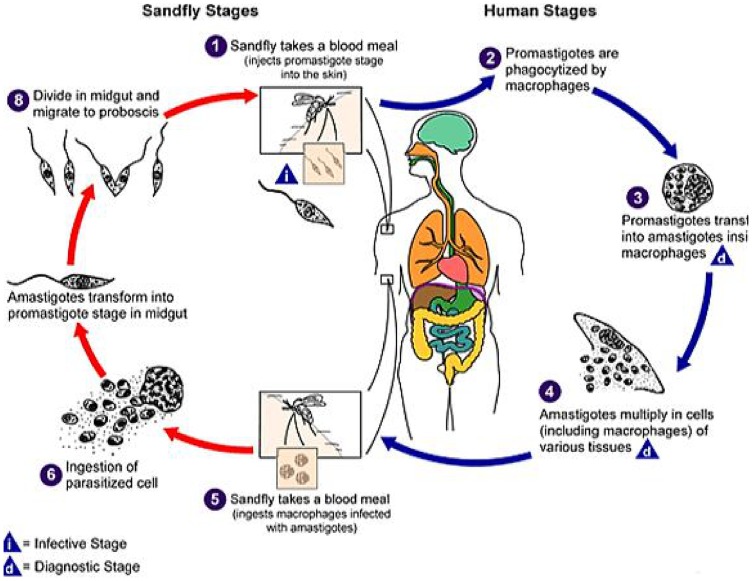
Life cycle of leishmania parasite [20].

The life cycle of the Leishmania parasite is completed in two hosts, a vertebrate host and an invertebrate host (phlebotomine sandfly) [20]. The parasites exist in two main morphological forms: the amastigotes in vertebrate hosts and promastigotes in invertebrate hosts [19]. The life cycle starts when the sandfly injects the infective stage (i.e., promastigotes) from its proboscis during blood meal. The promastigotes are then phagocytosed by the host's macrophages and other types of mononuclear phagocytic cells. Promastigotes transform in these cells into amastigotes. The mononuclear phagocytic cells lyses and then the amastigotes infect other phagocytic cells. The adult female sandfly is a bloodsucker, and when the fly bites a host infected with *Leishmania*, the amastigotes are ingested along with the blood meal. In the sandfly, amastigotes develop into promastigotes in the gut, and then migrate to the proboscis and the cycle continues when the sandfly injects the promastigotes into the skin of the host during blood meal [19], [20]. The life cycle of the *Leishmania* parasite is indicated in Figure 1. Transmission of *Leishmania* parasites can be zoonotic (i.e., from animals such as dogs and rodents to humans) or anthroponotic (i.e., from infected humans to non-infected humans) [20].

### 1.4. Vector ecology

There are about 30 species of phlebotomine sandflies known to transmit leishmaniases [26]. *Phlebotomus orientalis*, *Phlebotomus martini*, and *Phlebotomus celiae* have been confirmed as vectors of VL in Ethiopia [6], [25], [27], [28]. The ecological preference of these flies differs: rainfall, humidity, temperature, soil type and moisture content, and land cover type are significantly associated with the distribution pattern of these sandflies, although no universal pattern has been established so far [27], [28]. Changes in temperature, rainfall, and humidity can have strong effects on vectors and reservoir hosts by altering their distribution and influencing their survival and population sizes [5].

In the endemic areas of Ethiopia, two distinctive ecologic settings have been described. In northwestern foci, the principal vector, *P. orientalis*, is found in association with black cotton-clay soils and acacia forests. In southern foci, where *P. martini* and *P. celiae* are believed to transmit the disease, termite hills are thought to provide resting and possibly breeding sites for sandflies [28]. However, the Libo Kemkem and Fogera districts, which are affected by the recent epidemic, differ from both of the classic ecologic settings. These areas are located at an altitude of around 1800m, are substantially cooler, and have different vegetation from other endemic areas in the lowland areas [15], [22]. The vector species in this area has yet to be identified. However, the most likely vector inhabiting this area is believed to be *P. orientalis*.

### 1.5. Distribution

**Figure 2:**
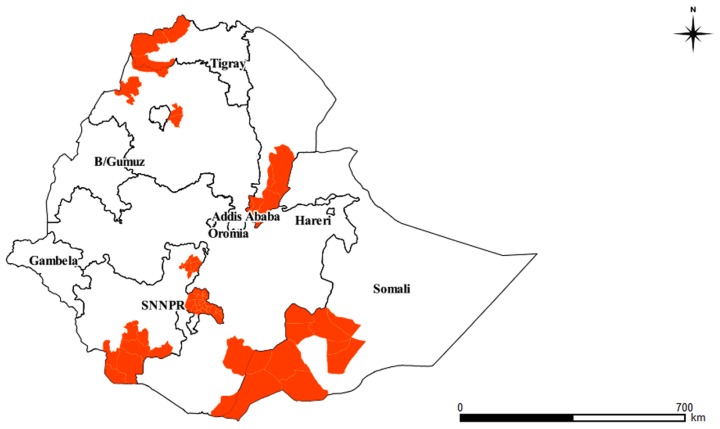
VL endemic foci in Ethiopia.

VL is reported to be widespread over the arid and semi-arid parts of the country. Cases of VL have been reported from six regions (Tigray, Amhara, Oromia, Southern Nations and Nationalities People's Region (SNNPR), Somali, and Afar regional states), and serologically positive cases were reported from Gambela and Benshangul Gumuz regional states [29], [30]. The most important endemic foci include the Metema and Humera plains in northern Ethiopia, the Omo plains, and the Aba Roba focus and Weyto River Valley in SNNPR. The disease was also reported from the Moyale area and Genale river basin in the Oromia regional state, Afder, and Liban zones in Ethiopia's Somali region, and the Awash Valley in the Afar regional state [6], [12], [14], [31]. Summary of VL foci in Ethiopia is detailed in Table 1 and illustrated pictorially in Figure 2.

**Table 1:** Summary of VL foci in Ethiopia.

Region	VL endemic districts/foci	Parasite involved	Vector involved	Reference
SNNPR	Omo: Omo plains	*L. donovani*	*P. orientalis*	[11], [35]
	Konso: Segen valley, Weyto valley	*L. donovani*	*P. martini*, *P. celiae*	[12]
	Sidamo: lake abaya, Dawa valley, Galena valley	*L. donovani*		[6]
Oromia	Moyale: Genale valley, Negele borena (Liben district)	*L. donovani*		[6], [31]
Somali	Afder	*L. donovani*		[14]
	Liban	*L. donovani*		[14]
	East Imey			[46]
Tigray	Humera plains: (Kafta Humera and Tsegede, Tahtay Adiabo districts)	*L. donovani*	*P. orientalis*	[25]
	Shiraro district			[46]
	Raya-Azebo			[30]
Amhara	Metema: (Armacho district)	*L. donovani*	*P. orientalis*	[25]
	Libo Kemkem	*L. donovani*, *L. infantum*	*P. orientalis*	[16]
	Fogera	*L. donovani*	*P. orientalis*	[6]
Afar	Awash valley: Mile, Dubty and Asayta	*L. donovani*	*P. orientalis*	[41], [42]

### 1.6. Risk factors

Different risk factors are known to engage in the epidemiology of VL. In endemic areas, more cases occur in younger age groups as they have yet to develop the acquired immunity. In East Africa, approximately 65% of VL cases are found in children less than 15 years old [18]. However, in outbreaks or areas where the disease has recently been introduced, all age groups are susceptible and most cases occur in groups that have regular contact with sandfly habitats [32]. Males are more predisposed to develop the disease as they are usually engaged in outdoor activities, which will make them more accessible to the sandfly bite [6].

Poor protein, energy, iron, vitamin A, and zinc nutritional status increase the risk of VL manifestation [19]. Malnourished children, who often suffer simultaneously from associated diseases such as tuberculosis, respiratory and/or intestinal infections are particularly vulnerable [5], [18]. A study conducted by Mengesha [33] indicated a significant association between severe malnutrition and intestinal parasitic infection, and VL. Malnutrition and intestinal helminth infection down-regulate the Th1 cellular immune response. Down-regulation of Th1 cellular immune response confers susceptibility to VL. Studies show that periodic regular deworming reduced malnutrition significantly, and supplementation of micronutrients such as zinc and iron, as well as vitamin A, improve malnutrition and enhance Th1 cellular immune response. Thus, periodic deworming and micronutrient and vitamin A supplementation together may reduce the risk of symptomatic VL [34].

Poverty increases the risk for VL. Poor housing and poor sanitary conditions may increase sandfly breeding and resting sites, as well as their access to humans. The flies are mainly attracted to crowded housing, as these provide a good source of blood-meals [5]. Sleeping outside or on the ground may increase the risk of infection. This is especially more evident in nomadic populations and in men who work in agricultural or pastoral settings due to increased time spent outdoors and thus higher exposure to the sandfly. Part of a community that lives and/or has frequent contact with acacia trees and termite hills are at increased risk, since acacia trees and termite hills are common breeding and resting sites for certain species of sandflies [5], [14].

Epidemics of leishmaniasis are often associated with migration and the introduction of non-immune people into areas with existing transmission cycles [19]. In northwestern foci, VL epidemics are associated with migration and the movement of non-immune workers into the VL-endemic extensive farm lands [5]. Workers from the non-endemic highlands are often victims of VL in these foci.

A recent study conducted by Bashaye et al. [22] investigating risk factors associated with the outbreak in Libo Kemkem identified dog ownership to be a risk factor. In Ethiopia, culture and tradition of inhabitants favours the keeping of dogs in urban and rural households often in close association with the family and farm animals. Almost all livestock owners and urban dwellers keep at least one dog to safeguard their properties from wild carnivores and thieves. These, and other, socioeconomic realities are considered to be conducive to the maintenance and further propagation of the disease.

### 1.7. Diagnosis and treatment

Currently, clinical diagnosis and the rK39 rapid dip-stick test (RDT) are used to diagnose VL in health centers, and clinical cases that are negative with rK39 RDT are referred for further diagnosis using the direct agglutination test (DAT) and/or lymph node or bone marrow aspirate at district or zonal hospitals. The first line treatment for VL in Ethiopia is sodium stibogluconate (SSG), and the second line treatment to be used in cases of toxicity, relapse, and treatment failure is amphotericin B [6].

## The Changing Epidemiology of VL in Ethiopia

### 2.1. Historical perspective

The lower Omo plains, in the southwestern foci, are the oldest known VL focus in Ethiopia, first identified in the 1940s [8]. Southwestern foci include the Omo plains, the Aba Roba focus, and the Weyto River Valley. In the 1970s, an extensive survey was conducted in this area [11], [35]. These authors reported sporadic endemicity of VL in these foci, which is characterized by widespread infection and low incidence affecting mainly children. Most of the population in this area has been exposed to the disease and acquired immunity. Fuller et al. [11] reported a prevalence of 64% using the leishmanin skin test. The Segen river (Aba Roba focus) and the adjoining Woytu river valleys are areas that exhibit a huge microfocal variation in burden of VL and *L. donovani* infection. The unique human adaptation and settlement patterns of the communities in these areas and the very focal nature of sandfly habitats are believed to be the major factors for the local variations in the burden of VL in the valleys [32]. The Aba Roba focus is especially one of the foci with high VL endemicity and high population immunity (a significant proportion of *Leishmania-*infected individuals does not develop clinical illness but shows elevated antileishmanial antibodies), with 36.4% testing positive with the leishmanin skin test [12]. The leishmanin skin test (LST) is used as an indicator of cell-mediated immunity against *Leishmania* parasites. It is believed that the development of a positive LST correlates with long-lasting protection against leishmania infection [36].

The other most important VL foci in Ethiopia are the Metama and Humera plains (northwestern foci) [10], [31], [37]. These lowland areas host the largest VL foci in Ethiopia and are adjacent to VL endemic areas of Sudan [38]. Before the 1970s, only sporadic cases of VL were known to occur in this area; however, a marked increase in VL cases occurred during the 1970s. In the Metama and Humera plains, the disease is particularly associated with migration of non-immune labourers from the surrounding highlands to the extensive agricultural farm lands of the Humera and Metema lowlands [39]. The marked increase in cases of VL in the 1970s in this area is also believed to be associated with the migration of workers from non-endemic highlands to intensive agricultural farm lands [37], [39], [40]. Several outbreaks have occurred in these foci; the most recent one that started in 1995 appeared to be more serious, claiming the lives of about 100–200 temporary farm labourers [25]. In the Humera and Metema plains, more than 2,500 cases of VL were being treated each year since 1998/1999.

In the lowlands of the northeastern Rift Valley of Ethiopia (Awash Valley), VL occurs with high population immunity. However, active case finding surveys by Fuller et al. [41] and Ali et al. [42] have not documented any confirmed cases of VL. On the contrary, leishmanin skin test rates remain high in this area. Fuller et al. [41] reported leishmanin rates of 59.5% among Afar pastoralists in this area. Recent surveys conducted by Ali et al. [42] in the lower Awash Valley have documented an average leishmanin skin test rate of 40%. No recent clinical case or suspects of VL were apparent in this area and thus, subclinical cases expected to make the bulk of the infections. There is no firm evidence as to why clinical cases of VL are not occurring in any great numbers.

The changing epidemiology of VL is evident in Ethiopia; the Libo Kemkem and Fogera districts, which are affected by the recent epidemic, differ from the known epidemiological pattern. Over the last decades, almost all cases and outbreaks of VL were reported from arid and semi-arid parts of the country. However, the Libo Kemkem and Fogera districts, where the recent outbreak occurred, are highland areas [15], [16]. The outbreak began in one peasant association in 2003, with cases peaking in 2005 and occurring mainly in the Libo Kemkem and Fogera districts. These areas have eventually become a low-incidence endemic area by 2007 [16]. This outbreak has claimed 120 lives among 2,450 primary cases that occurred, and it is the first known outbreak report of VL from the Ethiopian highland [22]. The parasite was thought to have been introduced by agricultural labourers returning from seasonal work in the Humera-Metema lowland areas. High prevalence of positive leishmanin skin tests were found in returned migrant workers [38].

### 2.2. Current situation

**Figure 3:**
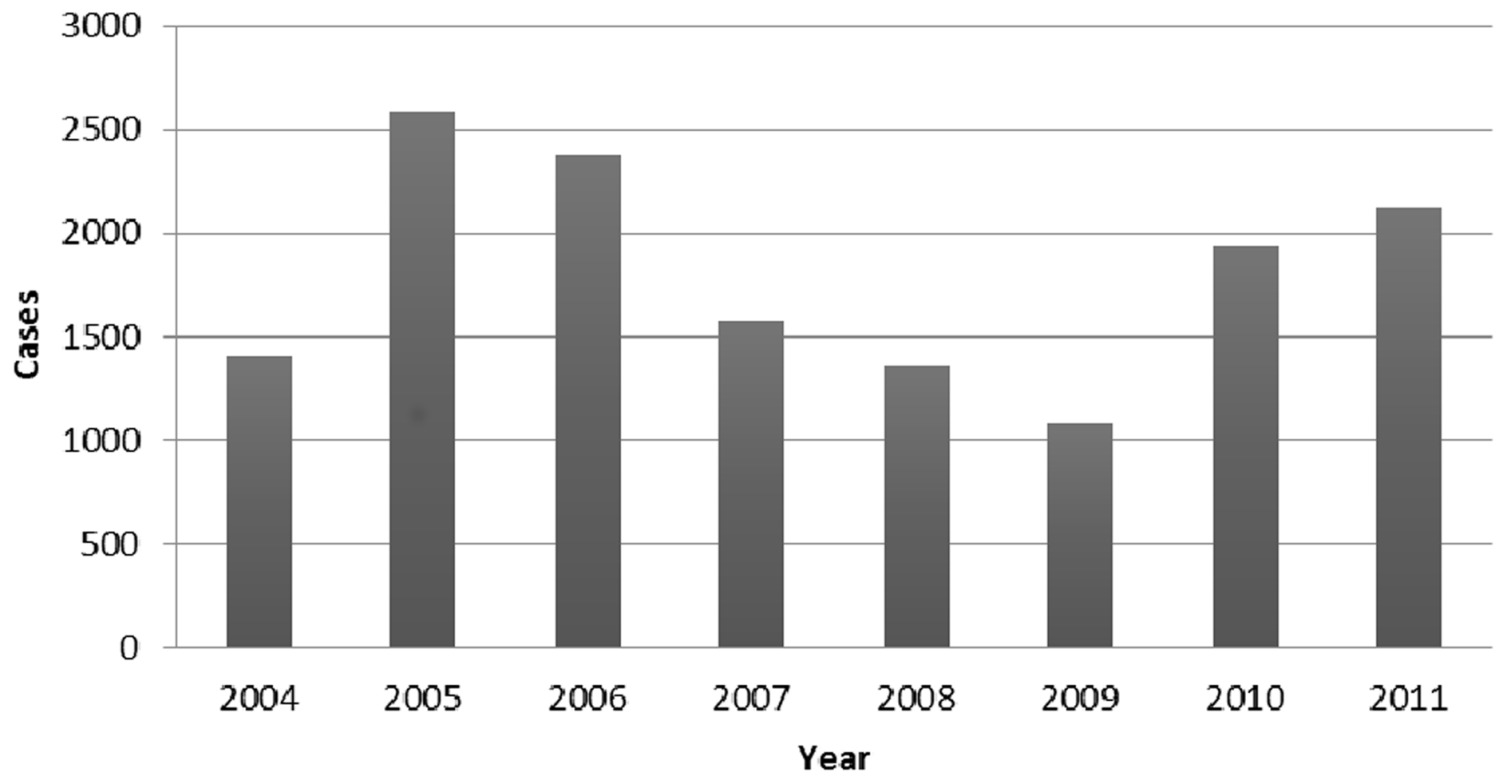
Trends of VL cases reported and treated by the MoH [46], [52].

There is a dearth of recent information on the distribution and burden of VL in Ethiopia, as there is a poor reporting system of the cases. According to the MoH [43], the annual burden of VL in Ethiopia is estimated to be between 4,500 and 5,000 cases. Unfortunately, this data is unlikely to give a true reflection of the field condition, as the health facilities where this disease is evident are not well equipped to identify VL cases. The trend of VL cases reported and treated between 2004 and 2011 is illustrated in Figure 3. In the Ethiopian practical context, it is highly likely that the VL burden might be much more than what is reported, because of the considerable migration of labourers from the highlands to the VL endemic lowlands, and foreseen HIV/AIDS epidemics in the endemic areas. Recently, Tsegaw et al. [30] developed a risk map of VL in Ethiopia. These authors estimated the total population at risk to be 3.2 million, based on the national population census of 2007. According to the risk map, an estimated 375,633 km^2^ (33%) of the country's landmass in northeastern, northwestern, western, and southeastern parts of the country is highly suitable for VL.

Given the increasing mobility of people and higher HIV–VL co-infection, there is an impending potential for VL to spread to the vast and highly populated areas of the country, since the distribution of the sandfly, a vector of VL, is believed to be more widespread than the disease.

### 2.3. Factors influencing the changing epidemiology

#### 2.3.1. Population movements

Epidemics of VL are often associated with migration and the introduction of non-immune people into areas with existing transmission cycles [19]. The changing epidemiology of VL in northern Ethiopia could most probably be linked to this factor. The outbreak of VL, which has occurred on the border between Kenya, Somalia, and southeastern Ethiopia in 2001 was also associated with migration of *Leishmania* infected population seeking food [14]. Population movements may also spread the disease, with the return of infected migrants to non-endemic areas. The source of infection for the recent outbreak in the Libo Kemkem and Fogera districts was also hypothesized to be migrant agricultural labourers returning to their villages from VL endemic areas [16], [22].

#### 2.3.2. Environmental and climatic changes

The occurrence and distribution of VL is strongly affected by environmental and climatic factors. Changes in temperature, rainfall, and humidity and other climatic factors have the potential to alter the geographic range of VL and its vectors [5]. In most endemic regions, VL is characterized by a patchy distribution, with discrete transmission foci. Microecological conditions affect the distribution of the vector, parasite, and the reservoir host by altering their establishment and survival [19]. A study was conducted in Ethiopia to identify environmental parameters influencing the geographical distribution of VL [30]. According to this study, annual average temperature shows a high leverage on VL occurrence (33%), and soil type is the second most important determinant (27%). Environmental changes that can affect the epidemiology of VL also include unplanned urbanization, deforestation, and the incursion of agricultural farms and settlements into forested areas [19], [30].

#### 2.3.3. Malnutrition and HIV co-infection

VL is an opportunistic disease. Immune suppression mainly due to malnutrition and co-infection with the human immunodeficiency virus (HIV) are among the factors believed to contribute to (re-)emergence of VL in Ethiopia [30], [44], [45]. The spread of HIV/AIDS to rural areas where VL is endemic has resulted in a progressive increasing overlap between the two diseases. Poor nutrition, HIV, and intestinal parasitic infections play a role in influencing the course of the disease by compromising patients' immunity [33]. The number of VL cases associated with immunosuppression has increased regularly over the past 15 or so years. According to a study conducted by Mengesha [33], among 403 adult VL patients examined in Northwest foci 385 (95.5%) were malnourished.

## Current Challenges

Probably the biggest challenge in the control of VL in Ethiopia is its association with HIV/AIDS, coupled with the seasonal migration of labourers to and from endemic areas. In northwest Ethiopia, up to 23% of VL cases are HIV co-infected in 2008, far higher than anywhere else in the world. The real burden is likely to be higher, as only 17% of VL cases are screened for HIV in some facilities [46]. Furthermore, individuals who tested HIV positive were more than four times more likely to die than those who tested HIV negative [4]. HIV/AIDS and VL are locked in a vicious circle of mutual reinforcement. VL accelerates the onset of AIDS by infecting macrophages throughout the reticuloendothelial system, rendering the body more susceptible by stimulating replication of the virus. In turn, HIV spurs the spread of VL [47], [48]. According to Sulahian et al. [49], AIDS increases the risk of VL by 100–1,000 times in endemic areas.

Generally, the public health impact of VL has been grossly underestimated, mainly due to lack of awareness of its serious impact on public health development over the last many years. Endemic regions have been spreading further and there has been a sustained transmission cycle. The public health impact of VL often becomes more evident in areas that are remote, where health facilities are absent or undeveloped [15]. In these areas, most critical cases are neither treated nor reported, so they act as a reservoir of infection, passing on the parasite to non-infected people and keeping the transmission sustained.

## Future Needs

The epidemiology of leishmaniasis, especially that of VL, in Ethiopia is complex, and a number of key questions remain unanswered. The reservoir hosts for this disease are not well known and the principal transmission modes are not clearly defined. As a result, no sound control measure is implemented so far. Surveillance of active cases and treatment are the major activities conducted so far by the MoH to control VL. Efforts made by the MoH to control the disease are suboptimal when compared to the public health burden the disease poses. Thus, it is highly likely that VL burden will increase, caused, in part, by the increasing migration of labourers, climate change, and impaired immunity.

Measures to control transmission vary according to local epidemiology [50]. Leishmaniasis control usually relies on case management (case detection and treatment), vector, and reservoir control [19]. Early case detection and treatment are essential for both individual patients and for the community. The treatment outcome is better in VL patients who are treated in an early stage. Untreated VL patients act as a source of infection and therefore contribute to disease transmission in anthroponotic VL areas [44]. In East Africa, up to 50% of the patients with VL develop post-kala-azar dermal leishmaniasis (PKDL). PKDL is a sequel of VL that appears as a macular, papular, or nodular rash, usually on the face, upper arms, trunk, and other parts of the body months after VL has apparently been cured. People with PKDL are considered to be a potential source of VL infection [5]. Thus, detection and treatment of PKDL patients is also likely to be beneficial; however, the feasibility and impact of PKDL control should have to be properly evaluated.

Where transmission is intense, case management alone has little effect, and control of transmission is more important [50]. Interruption of the transmission can be done through vector and/or parasitic control. Controlling sandflies is an integral component of leishmaniasis control. Vector control has never been practiced in the control of leishmaniasis in Ethiopia, and no convincing study was conducted to determine the role of sandfly control in the transmission dynamics of VL. In the Indian subcontinent, where *Phlebotomus argentipes* is reported to be the vector of VL, vector control was found to completely control VL [44]. Sandflies are susceptible to the same insecticides as *Anopheles* mosquitoes, the malaria vector. In Ethiopia, there is a massive *Anopheles* mosquito control campaign, so sandfly control could be planned alongside the *Anopheles* mosquito control campaign. However, it is important to note that the vector of VL in the Indian subcontinent, *P. argentipes*, is restricted to areas in and around the home. The habitat preference of the major sandflies believed to transmit VL in Ethiopia should be known before going for vector control. VL control through sandfly control could be successful, especially in domestic and peridomestic transmission habitats. The use of insecticide-treated bed nets (ITNs) prevents VL and other vector-borne diseases. In Ethiopia, using bed nets was found to decrease the risk of VL [38]. The use of ITNs avoids human–vector contact and thereby decreases the chance to be infected and also decreases the likelihood that sandflies will feed on infected individuals. However, in Ethiopia, bed net distribution and insecticide spraying take place in the context of malaria control.

The other most important VL control strategy is reservoir host control, especially in areas where the principal way of transmission is zoonotic. In Ethiopia, there is no conclusive evidence whether the principal way of transmission of *Leishmania* parasites can be zoonotic or anthroponotic. Therefore, in-depth studies of the reservoir hosts and transmission dynamics are very crucial before going for reservoir host control. Dogs are the main reservoir of *Leishmania* parasite, especially *L. infantum* in zoonotic VL [44]. The efficiency and acceptability of zoonotic VL control based on surveillance and culling of infected dogs is increasingly being debated. Treating infected dogs is not an effective control strategy, as relapses are frequent and dogs can regain infectivity weeks after treatment, despite being clinically cured. Furthermore, culling of infected dogs is considered unethical [19], [44]. The alternative approach in reservoir host control is the use of deltamethrine-treated collars, which reduced the risk of reservoir host infection. In a study conducted in Iran, using deltamethrine-treated collars in dogs protects domestic dogs from *L. infantum* infections and also reduce the risk of *L. infantum* infection in children [51].

## Conclusions

The epidemiology of VL is complex, and its control is an increasing challenge. The control measures taken so far cannot hold out its surge. Meanwhile, there has been a concurrent change in the epidemiology of VL, and there is an increasing body of evidence to suggest that the parasite and the vector are more able to adapt to a new environment than previously thought, as exemplified by the recent outbreak in highland areas of the Libo Kemkem and Fogera districts. What is certain is that with the spread of HIV infection to rural areas and the large-scale labourers' migration to and from endemic areas, VL will continue to pose a major public health problem. In order to reverse the current VL burden, it is important to understand the transmission dynamics of the parasite and the habitat preference of the vector. Therefore, further studies should be conducted to identify the potential reservoir hosts and to understand the transmission dynamics, as well as the habitat preference of phlebotomine sandflies. Further study is also needed to define the effectiveness of ITNs in the control of VL in Ethiopia and the role of the malaria control campaign in the reduction of VL burden.

**Key Learning Points**

- VL is one of the diseases that pose a significant burden to public health development in Ethiopia.
- There is a change in epidemiology of VL in Ethiopia.
- A diversity of risk factors is involved in the transmission of VL.
- The distribution and burden of VL in Ethiopia is larger than previously thought.

**Top Five Papers**

1. Tsegaw T, Gadisa E, Seid A, Abera A, Teshome A, et al. (2013) Identification of environmental parameters and risk mapping of visceral leishmaniasis in Ethiopia by using geographical information systems and a statistical approach. Geospat Health 7: 299–308.
2. Ayele T, Ali A (1984) The distribution of viseral leishmaniasis in Ethiopia. Am J Trop Med Hyg 33: 548–552.
3. Gebre-Michael T, Malone JB, Belkew M, Ali A, Berhe N, et al. (2004) Mapping the potential distribution of *Phlebotomus martini* and *P.orientalis* (Diptera: Psychodidae), vectors of kala-azar in East Africa by use of geographic information systems. Acta Tropic 90: 73–86.
4. Alvar J, Bashaye S, Argaw D, Cruz I, Aparicio P, et al. (2007) Kala-azar outbreak in Libo Kemkem, Ethiopia: epidemiologic and parasitologic assessment. Am J Trop Med Hyg 77: 275–282.
5. Lyons S, Veeken H, Long J (2003) Visceral leishmaniasis and HIV in Tigray, Ethiopia. Trop Med Int Health 8: 733–739.
